# Improving diabetic patients’ adherence to treatment and prevention of cardiovascular disease (Office Guidelines Applied to Practice—IMPACT Study)—a cluster randomized controlled effectiveness trial

**DOI:** 10.1186/s13063-022-06581-6

**Published:** 2022-08-15

**Authors:** Adesuwa Olomu, Karen Kelly-Blake, William Hart-Davidson, Joseph Gardiner, Zhehui Luo, Michele Heisler, Margaret Holmes-Rovner

**Affiliations:** 1 Division of General Internal Medicine, Department of Medicine, Michigan State University, East Lansing, MI USA; 2 Center for Bioethics and Social Justice & Department of Medicine, Michigan State University, East Lansing, MI USA; 3 Department of Writing, Rhetoric, and American Cultures, Michigan State University, East Lansing, MI USA; 4 Department of Epidemiology and Biostatistics, Michigan State University, East Lansing, MI USA; 5 Department of Internal Medicine, University of Michigan, East Lansing, MI USA

**Keywords:** Diabetes mellitus, Cardiovascular disease prevention, Medication adherence, Patient activation, mHealth intervention, Federally Qualified Health Care Centers, Shared decision-making, Health equity, Health disparities

## Abstract

**Background:**

Despite nationwide improvements in cardiovascular disease (CVD) mortality and morbidity, CVD deaths in adults with type 2 diabetes (T2DM) are 2–4 times higher than among those without T2DM. A key contributor to these poor health outcomes is medication non-adherence. Twenty-one to 42% of T2DM patients do not take blood sugar, blood pressure (BP), or statin medications as prescribed. Interventions that foster and reinforce patient-centered communication show promise in improving health outcomes. However, they have not been widely implemented, in part due to a lack of compelling evidence for their effectiveness in real-life primary care settings.

**Methods:**

This pragmatic cluster-randomized trial randomizes 17 teams in 12 Federally Qualified Healthcare Centers (FQHCs) to two experimental groups: intervention (group 1): Office-Gap + Texting vs. control (group 2): Texting only. Office-GAP (Office-Guidelines Applied to Practice) is a patient activation intervention to improve communication and patient-provider partnerships through brief patient and provider training in shared decision-making (SDM) and use of a guideline-based checklist. The texting intervention (Way2Health) is a cell phone messaging service that informs and encourages patients to adhere to goals, adhere to medication use and improve communication. After recruitment, patients in groups 1 and 2 will both attend (1) one scheduled group visit, (90–120 min) conducted by trained research assistants, and (2) follow-up visits with their providers after group visit at 0–1, 3, 6, 9, and 12 months. Data will be collected over 12-month intervention period. Our primary outcome is medication adherence measured using eCAP electronic monitoring and self-report. Secondary outcomes are (a) diabetes-specific 5-year CVD risk as measured with the UK Prospective Diabetes Study (UKPDS) Engine score, (b) provider engagement as measured by the CollaboRATE Shared-Decision Making measure, and (c) patient activation measures (PAM).

**Discussion:**

This study will provide a rigorous pragmatic evaluation of the effectiveness of combined mHealth, and patient activation interventions compared to mHealth alone, targeting patients and healthcare providers in safety net health centers, in improving medication adherence and decreasing CVD risk. Given that 20–50% of adults with chronic illness demonstrate medication non-adherence, increasing adherence is essential to improve CVD outcomes as well as healthcare cost savings.

**Trial registration:**

The ClinicalTrials.gov registration number is NCT04874116.

**Supplementary Information:**

The online version contains supplementary material available at 10.1186/s13063-022-06581-6.

## Administrative information

Note: the numbers in curly brackets in this protocol refer to SPIRIT checklist item numbers. The order of the items has been modified to group similar items (see https://urldefense.com/v3/__http://www.equator-network.org/reporting-guidelines/spirit-2013-statement-defining-standard-protocol-items-for-clinical-trials/__;!!HXCxUKc!zLkw_DQCt6R53fZ1y-ZCAHnKCwDW-5MjD0SIHX3-IepYAg7D04BjnJZ2lxT2OJF0IHjn8fq0auC6-is$).

**Table:** 

Title {1}	**Improving Diabetic Patients’ Adherence to Treatment and Prevention of Cardiovascular Disease (Office Guidelines Applied to Practice - IMPACT Study)- a Cluster Randomized Controlled Effectiveness Trial.**
Trial registration {2a and 2}	The ClinicalTrials.gov registration number is NCT04874116.
Protocol version {3}	**Version 2 of 11 5 2019**
Funding {4}	RO1HL149777 National Institutes of Health, National Heart, Lung, & Blood Institute
Author details {5a}	A. B. Olomu: Department of Medicine, Michigan State University, East Lansing, MI K. Kelly-Blake: Center for Bioethics and Social Justice & Department of Medicine, Michigan State University, East Lansing, MI W. Hart-Davidson: Department of Writing, Rhetoric, and American Cultures, Michigan State University, East Lansing, MI J. C. Gardiner: Department of Epidemiology and Biostatistics, Michigan State University, East Lansing, MI Z. Luo: Department of Epidemiology and Biostatistics, Michigan State University, East Lansing, MI M. Heisler: Department of Internal Medicine, University of Michigan M. Holmes-Rovner: Center for Bioethics and Social Justice & Department of Medicine, Michigan State University, East Lansing, MI
Name and contact information for the trial sponsor {5b}	National Heart, Lung, and Blood Institute, NIH
Role of sponsor {5c}	The sponsor did not participate in study design, submission of the grant, study implementation, nor in writing this manuscript. The sponsor has no authority over any of these activities. This study was supported by NHLBI/NIH RO1 HL149777-01A1.

## Introduction

### Background and rationale {6a}

The objective of this study is to decrease cardiovascular disease (CVD) risk for minority and low-income adults with type 2 diabetes (T2DM) by improving medication adherence. Despite nationwide improvements in CVD mortality and morbidity [1], CVD deaths in patients with T2DM are 2–4 times higher than among those without T2DM [2, 3]. A key contributor is medication non-adherence. Twenty-one to 42% of T2DM patients do not take blood sugar, blood pressure (BP), or statin medications as prescribed [4], contributing to increased morbidity and mortality [5]. Treatment and prevention regimens are complex, requiring innovative approaches to extending support beyond face-to-face clinic visits. Mobile health (mHealth) interventions hold promise as a scalable support strategy. However, research increasingly suggests that mHealth alone may not change behavior enough to improve health outcomes [6, 7]. In our pilot study to improve CVD care for patients with T2DM, we found that text messaging alone did not significantly improve patient activation, self-management, or medication adherence over 4 months. However, text messaging plus enhanced patient-provider communication in the Office-Guidelines Applied to Practice (Office-GAP) Program improved adherence to refills and medications compared to text messaging alone at 4 months [8].

Office-GAP is a patient activation intervention to improve communication and patient-provider partnerships through brief patient and provider training in shared decision-making (SDM) and use of a guideline-based checklist. In a pilot study under the PI’s K-award, among minority and low income T2DM and CVD patients, Office-GAP improved medication use, BP control, CVD knowledge, SDM, and patient and provider satisfaction with their communication at 6 months [9–11]. However, improvements were not sustained. A second pilot suggested that adding mobile phone texting to Office-GAP may help translate initial patient activation into long-term effective self-management [12]. We now propose to test and rigorously evaluate in a cluster randomized controlled trial, the combined Office-GAP and texting intervention (Way2Health) compared to texting alone, to determine impact on adherence to CVD treatment and prevention regimens in minority and low SES patients with T2DM [8, 13]. Our long-term goal is to reliably improve and sustain adherence in this vulnerable population to prevent CVD and eliminate disparities in CVD morbidity and mortality.

## Objectives {7}

We hypothesize that the combined intervention will improve both patient adherence and clinical outcomes over 12 months. We will randomize 17 teams in 12 FQHCs, recruiting 378 adults with T2DM to either (1) Office-GAP + Texting or (2) Texting only. Our primary outcome is medication adherence measured using eCAP electronic monitoring and self-report. Secondary outcomes are (a) diabetes-specific 5-year CVD risk as measured with the UK Prospective Diabetes Study (UKPDS) Engine score, (b) provider engagement as measured by the CollaboRATE Shared-Decision Making measure, and (c) patient activation as measured by the Patient Activation Measure (PAM).

- Aim 1: Compare the effects of Office-GAP + Texting intervention (group 1) with Texting only (controlling for attention) (group 2) in improving medication adherence, UKPDS risk score, provider engagement, and patients’ activation scores. Hypotheses:
  - H1: Group 1 patients will show significant improvement compared with group 2 at 6 and 12 months in the (a) primary outcome of medication adherence, (b) secondary outcomes of UKPDS score, (c) provider engagement, and (d) patient activation scores.
- Aim 2: Identify barriers and facilitators to adoption, implementation, maintenance, and spread using an integrated RE-AIM and Consolidated Framework for Implementation Research (CFIR) and to explore potential mediators and moderators of effectiveness.
- Exploratory aim 3: Determine whether there are differences in cost and health care utilization between groups 1 and 2 using the Micro-Costing Questionnaire and The Health Services Utilization Form.

## Trial design {8}

### Design

Our trial design is a pragmatic cluster-randomized trial of the test of equality between two groups. We test for equality of effects in two parallel intervention groups (1) Office-GAP + Texting and (2) Texting only, at 6 and 12 months and for change from baseline. The null hypothesis of all two-sided tests is the equality of effects between the two intervention groups. This pragmatic cluster-randomized trial randomizes 17 practice teams to two experimental groups: intervention (group 1): Office-Gap + Texting; control: Texting only (group 2). We plan to enroll at least 22 patients per team, with the target of having 320 evaluable patients for analysis. Because intact teams are randomized to treatment condition, and not individual patients, statistical analyses of outcomes in patients require accounting for intra-cluster correlation (“between subjects”) and serial correlation (“within subjects”) in repeated measures at baseline, 6 months, and 12 months.

**Fig. 1:**
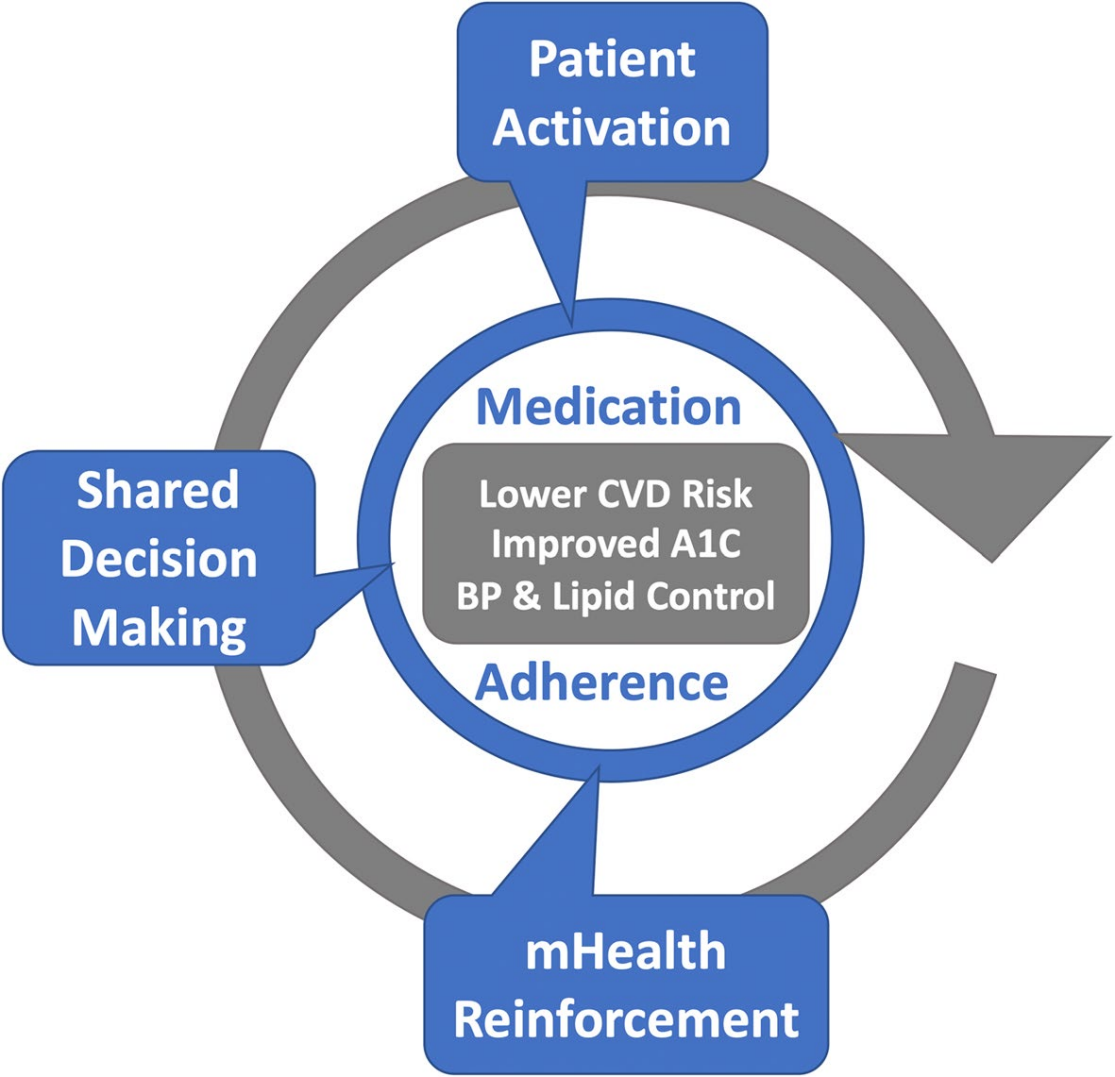
Combined interventions form a feedback loop

Two theoretical frameworks guided our intervention development, measures, and proposed analysis. The first, Self Determination Theory [14–16], suggests that effective interventions need to encourage patients to articulate their own values and goals (autonomy), agree that recommended behaviors correspond with these intrinsic values and goals (autonomous motivation) and have confidence in their ability to execute the targeted behaviors (competence), and second, Social Cognitive Theory [17, 18], which builds on the importance of understanding the quality of a health decision and of developing intrinsic motivation to change behaviors. It emphasizes patients’ self- efficacy in their ability to execute specific tasks (such as medication taking and involvement in treatment decision-making). Enhancing self-efficacy improves physiologic outcomes [12] and functioning [19]. We propose an intervention involving three interactions, forming a positive feedback loop (Fig. 1) [8].

The first is a patient-activation intervention in which patients are introduced to the evidence guiding their treatment plan and self-management. The second is the clinical encounter as a site for shared decision-making (SDM), between patients and providers, scaffolded by a checklist. Together, these first two steps constitute the Office-GAP intervention [9–11]. The third component is a mobile health-messaging program (Way2Health) that provides patients with ongoing between visit support, information, and a means to contact their provider between visits. Unlike stand-alone mHealth interventions, the Office-GAP Program is a patient activation intervention (PAI) that educate patients and providers in SDM. The combination of Office-GAP + mHealth provides a positive feedback loop [8].

## Methods: participants, interventions, and outcomes

### Study setting {9}

We are recruiting subjects from 12 FQHCs serving minority and low-income populations in Michigan, USA [20]. FQHCs are federally qualified, community-based, and patient-directed health centers serving low-income and underserved populations. They offer access to comprehensive care regardless of a patient’s ability to pay. These clinical sites are in urban, suburban, and rural areas of the state.

#### Sample

We will recruit a total of 378 patients with the goal of having 320 evaluable participants, self-identified men, and women, with T2DM that is not well-controlled (our standard will be a score for the HbA1c test 8 or higher than 8).

### Eligibility criteria {10}

#### Inclusion criteria

Patients: aged >18 at participating FQHCs who (1) have a diagnosis of T2DM with HbA1c >8, with or without CVD; (2) are taking at least one prescribed medication for BP, diabetes, or cholesterol management; (3) are able to provide informed consent; (4) are able to read and speak English (grade 6 reading level); and (5) have a cell phone with texting (provided by our study for any enrolled patient who does not have one). Providers: All providers in the participating clinics.

#### Exclusion criteria

(1) Medical record documentation of cognitive impairment, dementia, or psychosis; (2) plans to leave the area prior to study completion; and (3) participating in another behavioral diabetes intervention.

### Who will take informed consent? {26a}

A research assistant (RA) from our study team will conduct informed consent for patients at their initial group visit. We have three RAs, one in each of the three geographical areas where our clinical sites are located.

### Additional consent provisions for collection and use of participant data and biological specimens {26b}

This trial does not involve collecting biological specimens for storage.

### Interventions

#### Explanation for the choice of comparators {6b}

The comparators in this trial are clusters in which we implement a shared-decision making protocol consisting of a group visit in which patients learn tools to increase their skills and engagement in their care (“patient activation”) followed by a checklist used by providers, and patients to guide discussions during provider visits (the Office-GAP protocol) plus text messaging or text messaging alone. These options represent our approach to implement shared-decision making with text messaging as a mechanism of reinforcement in the Office-GAP group compared to text messages without a shared decision-making intervention. In our pilot study, patients’ medication adherence improved significantly with both Office-GAP + text messaging and with text messaging after four months. In the current study, we extend the intervention to 1 year to evaluate the impact of the arms on both medication adherence and cardiovascular risk score.

We hypothesize that the combination of Office-GAP plus text messaging for reinforcement will produce significant improvement in cardiovascular risk scores over 12 months. We also hypothesize that text messaging will improve patients’ cardiovascular risk at 3 and 6 months but will produce less improvement over 12 months than Office-GAP + text messaging. This hypothesis reflects our view that text messages are most valuable as reinforcement for a care plan established by the patient and provider through shared decision-making rather than as a substitute for a care plan.

#### Intervention description {11a}

Patients in either the intervention or control group will receive medical treatment-as-usual from the participating clinics, consisting of diabetes care, usual preventive care, and other medical treatment as needed (Table 1).

**Table 1:** Intervention components

	Physician and staff education model for all intervention groups	
	GROUP 1 PAI + Texting	GROUP 2 Texting only
**Baseline visit**	**Group visit** **• Office-GAP PAI** **• Way2Health Texting program** **• BP Check** **• Surveys and checklist use**	**Group visit** **• Way2Health Texting program Only** **• BP Check** **• Complete Surveys**
**PAI (group 1)**	**• Baseline evaluation and patients complete all study questionnaires** **• Introduction to CVD/DM and lifestyle changes** **• Discussion of benefits of using each cardiac/diabetes medication** **• Discussion of goal setting, shared decision-making, and patient engagement** **• Review of patients’ medications and completion of the** ***Office-GAP agreement*** **• Review of ACP Booklet** ***Living with Diabetes & Medication Adherence video*** **• Pre and post evaluation of the group visit**	
**Texting (Care4Life) (groups 1 and 2)**	**•** ***Medication Adherence Intervention*:** **Way2Health sends educational messages and quizzes on medication and adherence** **User responds to quiz** **•** ***DM Self-Management*** **Way2Health sends educational messages and quizzes on DM self-management** **User responds to quiz** **•** ***Blood Sugar Control Intervention*** **Way2Health sends educational messages about healthy blood sugar** **Way2Health sends blood sugar testing reminders** **User enters blood sugar readings and receives feedback on goals** **•** ***Blood Pressure Control Intervention*** **Way2Health sends educational messages about healthy BP** **Way2Health sends reminders to check and record BP** **User enters BP readings and receives feedback on goals** **•** ***Appointment Attendance Intervention*** **Way2Health sends appointment reminders** **User confirms appointment or reschedules**	
**6 and 12 months**	**-PAI Decision checklist** **-BP Check** **-Review of texting use** **- Surveys and exit interviews**	**-BP Check** **- Review of Texting use** **-Follow-up surveys** **-Exit Interviews**

##### Office-GAP intervention (patient activation intervention)

We have described the Office-GAP intervention in previous peer-reviewed publications [9, 10, 21]. Briefly, after recruitment, patients in the Office-GAP group will attend (1) one scheduled group visit (90–120 min; 4–6 patients at a time), conducted by the research assistants and (2) follow-up visits with their primary care providers within 1 month and then at 3, 6, 9, and 12 months after the group visit. At these follow-up visits, providers will complete the Office-GAP Checklist, which evaluates medication prescribing behavior. In addition, SDM and goal setting occurs between patient and provider. These follow-up visits are patients’ regularly scheduled visits with their primary care providers (PCPs) and not additional to their usual care.

##### At the group visit

We will obtain informed consent/HIPAA authorization from patients after introducing them to the study: Group 1: Office-GAP + Texting session: the group visit is a shared decision-making (SDM) activation session wherein patients learn self-management behaviors, communication skills, and use of decision support tools (DSTs). They also learn how to use the eCap electronic pill container to monitor medication adherence: learn to use Way2Health Texting service and confirm set up on their mobile phone. Group 2: For the texting only group: participants will learn how to use Way2Health Texting service and confirm set up on their mobile phone and learn how to use the eCap electronic pill container to monitor medication adherence. They will not experience the Office-GAP intervention. Both groups will also complete baseline study questionnaires during the group visit.

##### Office-GAP follow-up visit with providers

The Office-GAP Checklist is the core tool of our SDM intervention. A one-page checklist, this SDM tool outlines all evidence-based medications for secondary prevention of CVD in T2DM patients. It is an in-consultation decision support tool that helps engage the patient and provider to encourage and enhance an SDM process via discussion of medication and secondary prevention/lifestyle changes during an office visit. In this study, the Office-GAP Checklist will be completed by the physician with direct patient involvement during the office visits at 0–1, 3, 6, 9, and 12 months. Only group 1 patients will use this checklist during their office visits.

The Office-GAP Checklist also serves as a physician reminder at the point of care, because the physician records a checkmark for each medication: “Yes” (if patient is on the medication), “No” (if patient is not), or “Does not apply to me because….” A reason for exclusion of a medication is also provided (patient is not eligible for the medication, has a contraindication to its use, or the patient and physician have identified an alternative due to side effect or cost concerns). Details about the next appointment and any secondary prevention plan changes are also listed on the form. At the end of the visit, both patient and physician sign the form to confirm that they have reviewed the checklist. The patient receives a copy of the checklist to take home and a copy is retained in the patient record. All providers in the intervention teams receive a brief physician education in Office-GAP tool use and communication skills.

Educational tool literacy standards: Office-GAP has been rigorously adapted for use with low health literacy patients. All study materials are grade 6 reading level.

##### Mobile DM self-management texting intervention (Way2Health)

During group visits, all patients in both groups will be taught how to send and receive text messages on their phone. The Way2Health service does not require patients to use a special app. Way2Health engages patients in two ways: (1) Patients receive daily Way2Health messages appropriate to their diagnosis and medications (e.g., BP, blood glucose, medication) and appointment reminders throughout the study. They also receive informational and educational texts. (2) Patients respond to prompts that RAs monitors. In addition, they receive messages to contact their provider’s office with questions or concerns throughout the study via texting. Patients will receive additional diabetic modules that follow the standard for diabetes education for the rest of the 12 months once the initial 15-week program is complete. We will use the Way2Health texting program to encourage T2DM patients to maintain communication with their providers, improve medication adherence, and other secondary prevention and self-care between visits. Daily messages may take the form of reminders, prompts, education, or reinforcement. Sample message texts include “Did you take your medications today?” and “How many times did you check your feet for wounds this week?” Our pilot found the program to be usable and effective.

##### Provider and practice staff training

All intervention providers will participate in an interactive orientation which we have shown to promote behavioral change among health professionals. Providers will be asked to give their consent to participate in the study during this session. Provider/Staff Educational module: The training session is 60–90 min [22]. The session is scheduled multiple times to meet providers’ needs. In our pilot Office-GAP study, we achieved 100% participation by providers. The training module, facilitated by PI and co-investigators, includes (1) brief presentations on effectiveness and cost-effectiveness of medical therapy and behavior changes in management of T2DM, blood pressure, and heart disease; (2) a hands-on practice session for providers to elicit patients’ preferences and values; (3) an introduction to the Office-Gap Checklist and best practices for using it during a patient encounter; and (4) review of the Smith evidence-based patient-centered method for establishing trust, communicating clearly, and engaging in goal-setting with patients [22]. Providers will have a chance to practice skills using Braddock [23] and Elwyn’s [24]) approach to role-playing to model office visits; and (5) a complete pre-and post-training survey (before and after the training session) to determine provider attitudes about the use of DSTs, SDM, and mHealth in their practice and an end of study semi-structured interview.

#### Criteria for discontinuing or modifying allocated interventions {11b}

We try to keep modifications and discontinuation to a minimum in this pragmatic trial. However, if a patient does not consent or withdraws consent from a component of the study, or if a caregiver takes over administration of medications, these may be conditions for disenrollment. Our pragmatic trial takes every opportunity to document any barriers to implementation such as interruptions in cell phone service, scheduled hospitalizations, family vacations, etc., without disenrolling.

#### Strategies to improve adherence to interventions {11c}

##### Intervention integrity

Following the framework of Borrelli et al. [25], treatment fidelity ensuring the reliability and validity of Office-GAP and Texting will be maintained by (1) Decision Support Tool (DST) design; (2) training providers; (3) training and monitoring RAs; (4) delivery of educational modules during the group visits and use of mobile phones and DSTs; (5) receipt of DSTs and educational modules; use of study operating procedure manuals; and (6) intervention skills will be monitored by the PI/Project Manager. Intervention content quality will be monitored at weekly research team meetings. These measures will help ensure successful transferability to other settings [26].

Mobile service engagement is monitored on the W2H dashboard by RAs who can see responses from participants; data management oversight in RedCap to ensure that scheduling of patient visit milestones are being met.

#### Relevant concomitant care permitted or prohibited during the trial {11d}

Patients must not be enrolled in another cell phone intervention or behavioral diabetes management program.

Other relevant concomitant care for cardiovascular disease or diabetes is not excluded.

#### Provisions for post-trial care {30}

Because of increased monitoring from the Office-GAP program and short text messages, we might detect more episodes of elevated glucose or BP. Patients will be told by the RA under supervision of the PI, an Internal Medicine physician, to contact their PCP for immediate intervention and management of any abnormal blood glucose or BP.

We do not anticipate any substantial psychological risks to be associated with participation in this study. There are no financial or legal risks associated with this study.

### Outcomes {12}

All data will be collected at baseline, 6 months, and 12 months (Table 2). Our primary outcome is medication adherence measured using eCAP electronic monitoring and self-report. Secondary outcomes are (a) diabetes-specific 5-year CVD risk as measured with UK Prospective Diabetes Study (UKPDS) Engine score; (b) provider engagement as measured by CollaboRATE Shared-Decision Making measure; and (c) patient activation measures (PAM).

**Table 2:** Measures table

Variables	Instruments	Methods	Analysis	Time
**Knowledge and attitude about patient-centered care, SDM, use of DSTs**	**Pre and post survey of providers** **Semi-structured interviews**	**90 min. CME interactive orientation sections with providers and exit interviews**	**Descriptive pre-post analysis** **Thematic analysis of transcribed interviews using Dedoose**	**Pre and post physician education** **End of study**
**Medication adherence**	**Medication Events Monitoring Systems ( eCap).** **Cronbach’s alpha=.0.85** **ARMS Survey**	**eCap upload to BRIC** **RA will administer form to patient pre/post intervention to evaluate treatment medication adherence**	**Linear or generalized linear mixed effects model and Beta regression to evaluate changes over time for groups 1 and 2**	**Baseline, 6 and 12 months**
**Patient Activation Measure Scores/Level**	**Patient Activation Measure (PAM) Cronbach’s alpha=.91**	**RA administers form to patient pre and post intervention**	**Linear or generalized linear mixed effects model for change overtime for groups 1 and 2**	**Baseline, 6 and 12 months**
**Self-Efficacy Scores**	**Stanford 6-Item Self Efficacy Scale** **Summary DM Self-Activities Measure (SDSCA)**	**RA administers form to patient pre and post intervention to evaluate change in self-efficacy**	**Linear or generalized linear mixed effects models for change over time for groups 1 and 2**	**Baseline, 6 and 12 months**
**Rate of use of aspirin/antiplatelets, beta-blockers, ACEI/ARBs, cholesterol assessment and treatment, DM medications**	**PAI agreement in chart** **Patient medication list** **Patient medical record** **Texting log**	**Chart audits to compare the rate of use of aspirin/antiplatelets, beta-blockers, ACEI, cholesterol assessment and treatment of DM**	**Linear or generalized linear mixed effects model for change overtime for groups 1 and 2**	**Baseline, 6 and 12 months**
**Rate of BP control** **Diabetic Control (HbA1c)**	**Digital sphygmomanometer , HbA1c assessment in the community lab using standard instrument**	**Three BP measurements, 1 minute intervals, using appropriate cuff Reliable point-of-care HbA1c test**	**Linear or generalized linear mixed effects model for change overtime for groups 1 and 2**	**Average BP and HbA1c at baseline, 6 and 12 months**
**Combined Outcome Measure for Risk Communication & Treatment Decision Making Effectiveness (COMRADE)**	**COMRADE questionnaire** **Cronbach’s alpha=.92** **CollaboRate SDM Measure**	**RA administers form pre/post intervention to evaluate satisfaction with shared decision-making**	**Linear or generalized linear mixed effects model for change overtime for groups 1 and 2**	**COMRADE use at baseline, 6 and 12 months**
**PAI + Texting use rate & acceptability rating by clinical staff**	**PAI agreement forms; Texting log; pre/post physician/staff surveys; physician/staff interviews**	**RA records and chart abstraction to determine PAI + Texting use** **Staff meeting and survey to explore adoption + practice diary entries**	**Determine rate of use of PAI +Texting** **Frequencies of responses to evaluation surveys**	**Chart Abstraction at 6 months. RA tracks rates throughout study**

#### Primary outcome

Medication adherence is defined by the adherence metric (ratio of the number of days in which a patient takes his/her medication as prescribed, to total number of days he/she is expected to take them in that period) [27, 28]. Since there is no ideal medication adherence measure, a multi-measure approach is often recommended in assessing medication adherence to maximize ecological validity [28]. A meta-analysis also showed that this approach, including using a self-report method, can increase sensitivity to non-adherence [28, 29]. The concomitant use of both objective and subjective measures will provide higher reliability and represents the most rigorous approach to assessing medication adherence [30]. We will measure medication adherence using the eCap electronic pill container: the most objective and valid method of assessing adherence [28, 31], and a self-reported adherence measures described below. eCap will record the number of times the pill bottle is opened daily, over the course of the study. As in other trials, patients will be given eCap to assess adherence to their most frequently taken CVD prevention medication (e.g., BP, blood sugar lowering or cholesterol-lowering medication) [32], as identified by the primary care physician (PCP). Studies have shown that adherence to one medication parallels adherence to others [33]. Any medications taken during emergency room visits or hospitalizations will be obtained from patient reports and chart abstraction of patient medical records to avoid erroneously penalizing patients when the eCap is not used. Such days will be removed from the denominator of the formula to compute adherence rates. Self-reported adherence will be assessed using Adherence to Refills and Medications Scale (ARMS) a 14-item scale with two subscales for taking medications as prescribed and refilling medications on schedule [34, 35].

#### Secondary outcomes

Secondary outcomes are as follows: (a) diabetes-specific 5-year CVD risk as measured with UK Prospective Diabetes Study (UKPDS) Engine score; (b) provider engagement as measured by CollaboRATE Shared-Decision Making measure; and (c) patient activation measures (PAM). The UKPDS score includes components we hypothesize will be improved by the intervention, including HbA1c, systolic blood pressure, cholesterol levels, and smoking status. Using a cardiac risk score allows us to quantify the cumulative impact of changes in multiple risk factors and translate changes in physiologic parameters to risk estimates that are meaningful to patients and policy makers [36]. Cardiac risk scores including the UKPDS Risk Engine have been successfully used as outcomes in multiple clinical trials [37–41] and validated in multiple populations [42]. A 1–2% overall change in risk can be considered clinically significant at a population level [43]. We will measure HbA1c using a portable Afinion AS 100 finger stick sample analyzer, which is accurate and has a coefficient of variation <5% as required by the National Diabetes Data Group [44].

### Participant timeline {13}

Our trial establishes the following timeline:

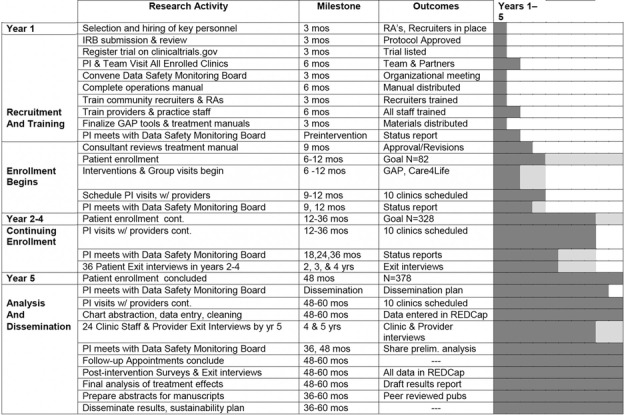



### Sample size {14}

#### Sample size assessments

##### Hypotheses

Statistically significant differences between groups are not expected at baseline (t=0). Our primary hypotheses concern the difference in differences (DIDs) at 6 months and 12 months, where we expect both clinically meaningful and statistically significant differences. Sample size assessments for the study are based on demonstrating 80% power to test the primary hypotheses in Aim 1.

Denote by *Y*_*hit*_ an outcome assessed at time *t* in the *i*-th patient in the *h-*th practice team. Generally, outcomes are assessed at baseline and at 2 additional time-points 6 and 12 months. We formulate our test of hypothesis using a generalized linear (mixed) model (GLMM) for the expected response: *μ*_*hit*_ = *E*(*Y*_*hit*_| **x**_*hit*_, **b**_*hi*_)where **x**_*hit*_are patient characteristics and **b**_*hi*_ are random effects to capture serial correlation within participant measures and clustering.

Minimally, let ${\mathbf{x}}_{hit}^{\prime}\beta ={\beta}_{0}+{\beta}_{1}{z}_{1}+\left({\beta}_{21}{t}_{1}+{\beta}_{22}{t}_{2}\right)+{z}_{1}\times \left({\beta}_{31}{t}_{1}+{\beta}_{32}{t}_{2}\right)$where *z*_1_is the indicator for group 1 with group 2 as referent, 2 indicators *t*_1_,*t*_2_, for time with baseline as referent and group by time interactions. Interpretation of parameters is shown in Table 3.

**Table 3:** Exemplar parameter interpretation in model

Study Arm	Time			Change from t=0	
	t=0	t=6m	t=12m	At t=6m	At t=12m
GROUP2	*β*_0_	*β*_0_ + *β*_21_	*β*_0_ + *β*_22_	*β*_21_	*β*_22_
GROUP1	*β*_0_ + *β*_1_	*β*_0_ + *β*_1_ + *β*_21_ + *β*_31_	*β*_0_ + *β*_1_ + *β*_22_ + *β*_32_	*β*_21_ + *β*_31_	*β*_22_ + *β*_32_
DIFF	*β*_1_	*β*_1_ + *β*_31_	*β*_1_ + *β*_32_	DID=*β*_31_	DID=*β*_32_

Hence the net difference (DID) at 12 month is *β*_32_. Our study is powered to test the hypotheses: (a) *H*_0_ : *β*_31_ = 0, (b) *H*_0_ : *β*_32_ = 0and jointly (c) *H*_0_ : (*β*_31_, *β*_32_) = 0. Two other design features are a serial correlation *ρ*_*W*_ between repeated measures over time, an intra-class correlation (ICC) *ρ* for clustering within practice team. We obtained plausible inputs for *r* and *ρ* from the literature and our own experiences [45–48]. In community intervention studies *ρ* is small (we use 0.002) but *ρ*_*W*_ is much larger (we use 0.30). The hypothesized adherence rates at 0, 6, and 12 months are 0.40, 0.42, and 0.42 in group 2 and projected to be 0.40, 0.62, and 0.65 in group 1. With 9 teams in the combined intervention group, 8 in the text alone group, and at least 22 patients in each team, the power to detect differences between groups at 6 months and 12 months is 80.8% and 90.4%, respectively. The power to detect the DID at 6 months is 71.4% and 83.1% at 12 months. Thus, our target sample size is a total of 320 patients. Because some attrition is expected over the 4-year period, we will recruit a total of 378 patients to account for attrition of 10% in YR=1, another 4% in YR=2, and another 2% in YR=3.

### Recruitment {15}

#### Recruitment and retention plan

**Fig. 2:**
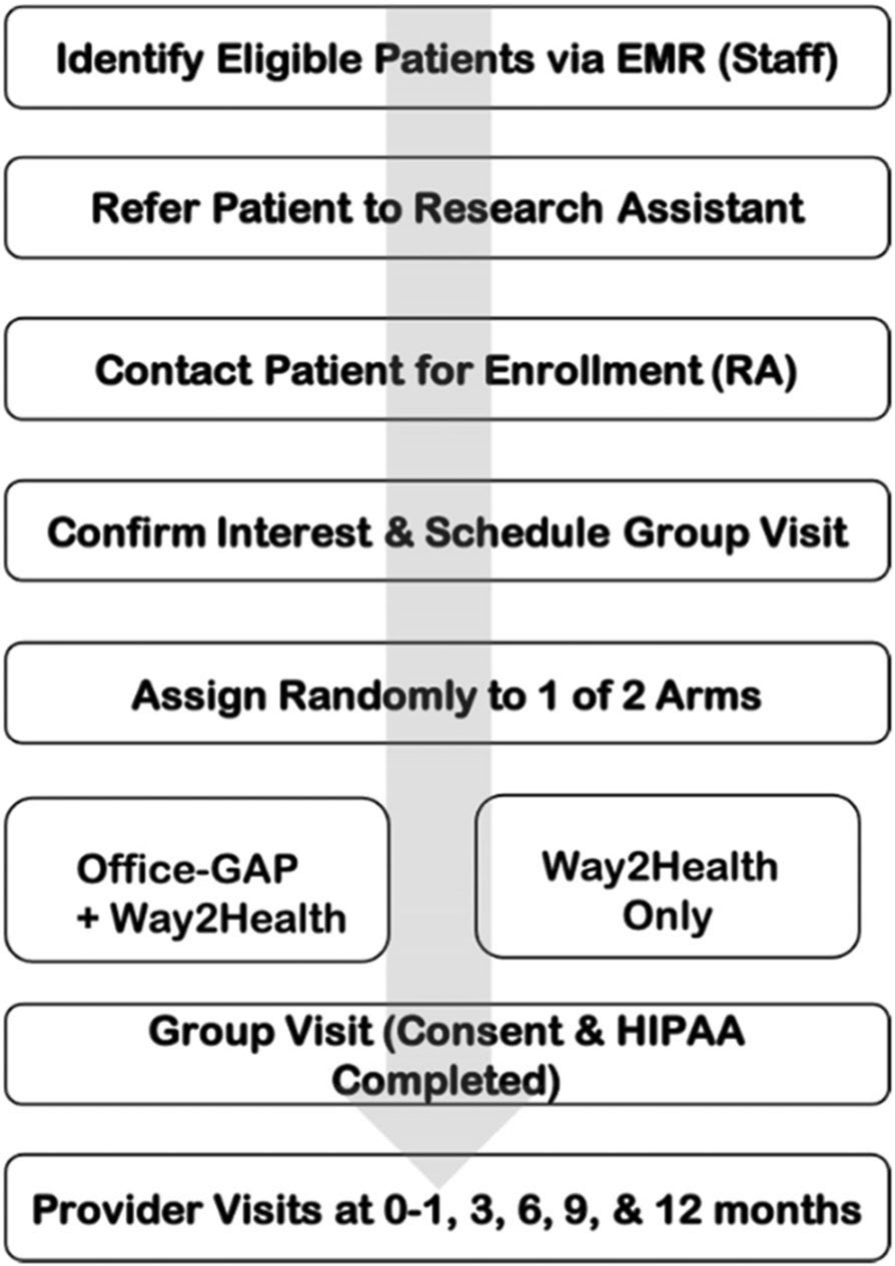
Patient flow

Patients will be recruited from participating FQHCs using a variety of recruitment strategies the PI has used successfully in her prior research at FQHCs (e.g., identification by EMR, patient flyers, working with clinic staff members and providers). Patient recruitment during the first 4 years of the study will proceed as follows (Fig. 2):

1. A web interface through EMR links patient information and scheduling information at the time of each encounter form generation. EMR queries the preloaded databases and/or registries to determine if there is a match for the study, based on diagnosis codes (ICD 10- E08.00).
2. At the visit to the primary practice where diabetes is on the problem list, EMR will flag the patient chart and indicate that the patient is eligible for the study. The practice staff will inform the patient about the study and direct the patient if interested to the research assistant (RA) for more information regarding the study. If the RA is not available, the practice staff will give the permission to call (PTC) form to interested patients to complete and sign for RA to pick up.
3. A patient’s primary care physician (PCP) can identify patients as having diabetes by adding Diabetic codes to their EMR problem list.
4. Patients that sign the PTC Form will be contacted by the RA either in the office or over the phone. The RA will briefly describe the project to the patient and if he/she is interested to participate, he/she will be scheduled for a group visit. The consent and HIPAA forms will be completed during the group visit. The RA or PI is responsible for answering questions and explaining the consent. Practice personnel will not be involved in obtaining consent.
5. The patient may consent to the study, including follow-up, or may decline. At the time of consenting, the patient will be asked for several ways they can be contacted for follow-up. The practice will retain one copy of the consent, one will be forwarded to data entry (see below) and then to the RA who will retain it in the study files, and a copy will be given to the patient. The patient’s response to the enrollment offer will be entered into EMR as their consent form version (consent declined, or consent). If the patient declines to consent to the study, the patient will follow up with their physician as usual.
6. Patients in clinics/teams randomized to group 1 will experience the patient activation intervention (Office-GAP) + medication adherence and life-style promotion intervention via Short Message Service on their cell phone (Way2Health).
7. Patients in clinics randomized to group 2 will only receive medication adherence and life-style promotion intervention via Short Message Service on their cell phone (Way2Health).

## Assignment of interventions: allocation

### Sequence generation {16a}

#### Randomization

We chose team-level assignment to unify all providers and patients at a team into a single intervention, minimizing risk of contamination. Seventeen teams from 12 clinics in 3 counties (with 4, 7, and 6 teams, respectively) agreed to participate in the trial. We first generated a uniformly distributed random variable (U) using a pseudo-random number generator in Stata and each team was allocated a random number. Then we stratified the teams by county, and the randomization procedure within each county followed these steps. In the county that had 1 team per clinic, the teams were sorted by U and the first half of the teams were assigned for group 1 and the second half for group 2. In the county that had 3 clinics with 2 teams and 1 clinic with 1 team, we sorted the teams within each clinic by their U values and in the 2-team clinics the first one was assigned to group 1 and the other to group 2; and the single-team clinic was randomly assigned to group 1 because U was less than 0.5 (which is a predetermined rule for assignment). Finally in the county with 3 teams in 1 clinic and 1 team in 1 clinic, all teams were sorted by U and the first 2 teams were assigned to group 1 and the last 2 teams were assigned to group 2. Overall, 9 teams were assigned to group 1 and 8 to group 2.

We chose this approach because it is realistic for providers and patients. This also helps the comparison of control and intervention groups to be more accurate because there is less chance of mixing between the two groups. In Michigan, FQHCs have similar numbers and types of providers (internal medicine, and family medicine) and nurse practitioners.

### Concealment mechanism {16b}

We do not randomize patients; we randomize clinics. Allocation is concealed during recruitment. We reveal to both patients and providers which arm the clinic has been randomly assigned to once they give consent to participate.

### Implementation {16c}

The statistician generated the allocation sequence, and all clinical staff were notified prior to enrolling patients. Because this is a cluster randomized trial, all patients at a particular team will be in the same arm. Patients are enrolled by the RA assigned to each clinic after signing the informed consent agreement.

## Assignment of interventions: blinding

### Who will be blinded? {17a}

After the 17 teams are randomly assigned to the two arms, neither trial participants nor the care providers can be blinded to the assignment due to the nature of the intervention. Neither the participants nor the researchers are blinded for this study.

### Procedure for unblinding if needed {17b}

The design is open label so unblinding will not occur.

## Data collection and management

### Plans for assessment and collection of outcomes {18a}

#### Primary outcome—Table 2

We will measure medication adherence using the *eCap electronic pill container*: the most objective and valid method of assessing adherence [31] and a Self-Reported Adherence measures described below. eCap will record the number of times the pill bottle is opened daily, over the course of the study. As in other trials, patients will be given eCap to assess adherence to their most frequently taken CVD prevention medication (e.g., BP or cholesterol-lowering medication) [32], as identified by the primary care physician (PCP). Any medications taken during emergency room visits or hospitalizations will be obtained from patient reports and chart abstraction of patient medical records to avoid erroneously penalizing patients when the eCap is not used. Such days will be removed from the denominator of the formula to compute adherence rates. *Self-reported adherence* will be assessed using *Adherence to Refills and Medications Scale (ARMS)* a 14-item scale with two subscales for taking medications as prescribed and refilling medications on schedule [34, 35].

*Secondary outcomes*—baseline, 6 and 12 mo. (a) Diabetes-specific 5-year CVD risk as measured with UK Prospective Diabetes Study (UKPDS) Engine score, (b) provider engagement as measured by CollaboRATE Shared-Decision Making measure, and (c) patient activation measures (PAM).

We will measure HbA1c using a portable Afinion AS 100 finger stick sample analyzer, which is accurate and has a coefficient of variation <5% as required by the National Diabetes Data Group [49].

#### Measurements

Cholesterol measurements will be obtained from patient charts at baseline, 6 months, and 12 months. Providers will order this test during patients’ visits. We will assess smoking status using a CDC questionnaire [50]. *Blood pressure* (BP) will be measured by the RA at baseline, 6 months, and 12 months for all enrolled patients using a validated automated device (*BpTru*), following AHA guidelines [51]. Three BP measurements will be obtained at 1-min intervals. Average systolic BP (*SBP*) and diastolic BP (*DBP*) will be recorded. Inadequate BP control will be defined as mean SBP > 130 mm/Hg or DBP > 80mm/Hg per 2019 guidelines for DM patients [52].

#### Surveys (Table 2)

Completed at 0, 6, and 12 months: (a) CollaboRATE Shared-Decision Making measure: CollaboRATE, a fast and frugal patient-reported measure of SDM process, contains 3 brief questions that patients complete following a clinical encounter [53, 54] and has inter-rater reliability of .86 at .001 significance. (b) Patient Activation Measure: The Patient Activation Measure (PAM) consists of 13 items that form a scale with a Cronbach’s alpha of .9 [55]. (c) Combined Outcome Measure for Risk Communication And Treatment Decision Making Effectiveness (COMRADE) [56] measures patient satisfaction with physician communication, and patient confidence in decisions made. This scale has good internal consistency (Cronbach’s alpha=.92); (d) Stanford Self-Efficacy Scale for Managing Chronic Diseases – 6-Item Scale with internal consistency of reliability of .91 [57]. (e) Summary of Diabetes Self-Care Activities (SDSCA) Measure, a multidimensional measure of DM self-management; with good internal and test-retest reliability [58]. (f) the Rapid Estimate of Adult Literacy in Medicine (REALM) [59], a widely used measure of health literacy [60] tailored to poor medication adherence [61].

#### Participant characteristics and potential confounders

A comprehensive list of participant characteristics will be collected, including socio-demographic characteristics (including sex, gender, age, etc.), medical history, comorbidities, and health behaviors. System Usability Survey (SUS) [62] used in our Office-GAP study and Way2Health Pilot study will ask about texting, computer, Internet use, and comfort when using a mobile phone and its features. Medications: medication names and their doses will be obtained. We will determine change in proportion of patients on aspirin, other antiplatelet medications, beta-blockers, cholesterol-lowering agents, and angiotensin converting enzyme inhibitors/angiotensin receptor blockers (ACEIs/ARBs). We will collect data on smoking cessation counseling and reduction in proportion of patients’ smoking.

### Plans to promote participant retention and complete follow-up {18b}

#### Retention plan

To maximize retention and prevent attrition, 0–1-, 3-, 6-, 9-, and 12-month follow-up assessments will be scheduled at the preceding visit (Fig. 2); follow-up reminders will occur via text messaging, calls, and mailings beginning 3 weeks prior to each visit. Patients will be compensated $20, $20, $20, $20, and $20 for attending group visit and the 0–1-, 3-, 6-, 9-, and 12-month follow-up visits respectively in the form of gift card, to cover transportation, parking, and time. At the 12-month visit, the control group (texting only) will be compensated $50 while the intervention group (Office-GAP + Texting) will receive $100 in the form of a gift card. Participants that complete an exit interview will receive an additional $40. Using these strategies in our Office-GAP studies with this population had >80% retention rate.

### Data management {19}

This study is using the Research Electronic Data Capture (REDCap) system for data entry, coding, security, and storage. Data will be entered by using electronic data capture forms, which will be created by the Biomedical Research Informatics Core (BRIC) from paper forms provided by the PI. The BRIC Data Manager will utilize standard quality assessment features within REDCap to monitor ongoing data quality: e.g., missing data for key variables, distribution of variable values, completeness of form data entry, etc. BRIC will design and deploy a programmatic utility to process eCAP data: downloading cloud data, cleaning, analytical structuring, and “number of days adherence” variable derivation. BRIC will maintain secure storage and backup of the downloaded eCAP data.

#### Data quality

The use of REDCap, which may include a Study-specific data dictionary, adaptive logic, and real time validation rules, with assistance from the BRIC informatics team results in a well-planned data collection protocol and quality assurance strategy.

#### Data sharing

The REDCap system also provides a standard export mechanism, which is easily exported to a variety of types of common statistical packages (SPSS, SAS, Stata, R/S-Plus). This allows the Principal Investigator (PI) to generate data that are truly independent of the method of data entry, thus generating usable, collaborative datasets for sharing and outcome analysis.

### Data monitoring plan

The Director of Biomedical Research Informatics Core will oversee and ensure implementation and monitoring of the data management plan and will be responsible for general oversight of BRIC research efforts and safeguards related to the protection of human subjects’ data. He will work closely with the BRIC Data Managers to ensure that the Informatics configuration, testing, and production processes are conducted appropriately to ensure security and confidentiality of data in the Study’s REDCap database. Data will be entered into the REDCap, by the Research Assistants (RAs) and will be independently verified by the Project Manager/another RA.

#### Data security

The REDCap system was designed specifically around HIPAA-Security guidelines. User access to data can be restricted at the form/survey level. In addition, variables may be designated as ‘identifying’, which excludes their inclusion in data exports. The MSU- REDCap server is housed in a facility which satisfies requirements for the storage of HIPPA data. Security staff patrols, RFID key card access, and audio/video monitoring protect the physical location of the server. The server is connected to a private segment of the BRIC network. The segment is protected through a firewall, which routes traffic and provides active intrusion detection and protection. All web-based information transmission is encrypted and uses Secure Hypertext Transfer Protocol (HTTPS), which employs a standard Secure Sockets Layer (SSL - 128 bit block size) encryption technology. SSL creates the secure connection, and HTTPS transmits the data securely. All subject data will eventually reside in a MySQL database server. Medication adherence data collected by using eCAP-fitted medication vials will also be collected in the study protocol. Servers hosting the REDCap database and eCAP data are maintained by the Biomedical Research Informatics Core (BRIC) at Michigan State University.

### Confidentiality {27}

The procedures for obtaining research material for the study include anthropometric and BP measurements, laboratory data (e.g., glycosylated hemoglobin {HbA1c}), medical records, interviews, and self-completed questionnaires. Research Assistants (RAs) that are trained and certified will obtain all data according to detailed study protocols. Data will be collected directly from enrolled study participants and medical records and used specifically for research purposes. Baseline data will be obtained during group visits only after the patient has signed the informed consent and the HIPAA form. Follow up data will be obtained at 0–1, 3, 6, 9, and 12 months after office visits with their providers. Only individuals officially assigned to the study team or data management team will have access to individually identifiable information about human subjects. This will include the PI, co-investigators, project coordinator, statistician, and research assistants. All individuals will have completed MSU’s IRB human subjects training and will be included on a staff listing of the MSU IRB. Patient survey information, initially collected using paper forms, will be entered into the REDCap database; EMR data will be abstracted using paper forms and later entered into the REDCap database by RAs; eCAP data will be uploaded into eCAP-Provider cloud storage by RAs and then securely downloaded by the BRIC Data Manager to a secure BRIC server.

Access to data in the REDCap database will be controlled by using user definitions and permissions, which exist within the REDCap administration module. The PI will be responsible for authorizing users and granting permissions for access to the data. Audit logs are created automatically within the REDCap application to capture the complete user history of database activity, enabling compliance to regulations of electronic records and signatures. eCAP (electronic cap for monitoring medication adherence) data will be downloaded from the eCAP provider’s cloud server by BRIC personnel and stored in de-identified format in the REDCap File Repository. The Study Statistician will access these data directly by logging in to REDCap. Daily and monthly backups of the REDCap and eCAP data will be made and retained on a server within the MSU-ITS computer data center with climate-control, motion-detection security, and swipe-card entry. REDCap variables designated as “personally identifying” will be excluded from export by using REDCap user-defined permissions. In addition, BRIC tracks the initial certification of IRB training for all REDCap users, and user access to REDCap is permitted only after documentation of certification is received.

### Plans for collection, laboratory evaluation, and storage of biological specimens for genetic or molecular analysis in this trial/future use {33}

See above in 26b. This study will not collect biological specimens for storage.

## Statistical methods

### Statistical methods for primary and secondary outcomes {20a}

#### Statistical analysis

Medication adherence will be assessed in individual patients by eCAP monitoring. Hypotheses will be tested at 6 months and 12 months.Tests are two-tailed at 5% level of significance. We compare group 1 patients to group 2 patients at baseline using, as appropriate, ANOVA-F-tests, chi-square tests, and non-parametric tests to assess equivalence of potentially confounding physiological and contextual patient characteristics. If substantive differences are found, they will be adjusted for in subsequent analyses by regression techniques, guided by the degree of dissimilarity rather than strict statistical significance [63]. Equivalence between the groups will be assessed on age, gender, education, race/ethnicity, use of statins, b-blockers, aspirin, and ACE1/ARB, blood sugar and lipid values, and team factors such physician experience.

##### Analysis of the primary and secondary outcomes

We adopt a regression-based approach to multivariable modeling that incorporates features of clustering of patients within the practice team and correlation over time in repeated assessments. Repeated measures ANOVA, or more apropos, generalized linear (mixed) models (GLMM) will be used [64]. Generally, outcomes are assessed at baseline and at 2 additional time-points 6 months and 12 months. By choice of link function connecting the expected patient outcome to covariates, GLMM can be applied to different types of outcomes (continuous, categorical, count, ordinal). They can incorporate random effects to capture serial correlation within patient measures, and clustering of patients within practice teams. Covariates are patient characteristics, some of which are time-invariant such as the aforementioned sociodemographic variables, and characteristics of the practice team. Key right-hand side variables in addition to the covariates are a single indicator for group, with control as referent, two indicators for time, with baseline as referent and group by time interactions. The GLMM allows us to formulate and test hypotheses on functions of the regression parameters: they include (i) point-in-time comparison between group 1 and group 2, (ii) time-averaged comparison between group 1 and group 2, (iii) within group comparison for change over time, and (iv) change in group 1 compared to the corresponding change in group 2 via difference-in-differences (DID). SAS Software (ver 9.4, Analytics 15.1 or higher) will be used for statistical analyses.

##### Analysis of the exploratory outcomes in Aim 3

Cost analysis will provide estimates for the value of resources used by each patient [65]. The value of resources not directly related to patient interaction, such as training costs and non-labor costs, will be allocated evenly across the intervention groups. Direct medical costs include expenditures for medical services and products usually paid for by the health system and consumer, including intervention costs (group visits, Texting), cost of medical care outside the interventions (use of hospital, emergency room, urgent care, outpatient visits, and prescription medications), and IT support (text messaging support). Direct non-medical costs include time and transportation to and from clinic visits and care provided by family members. Total cost per group at the end of the study will be computed by summing the costs for each patient. Mean cost per group will be calculated by dividing the total cost by the number of patients in each group. Research costs will not be included. Cost analysis will consider the perspective of the health system (FQHCs) and the consumer (patient). For costs analyses, we will follow the guidelines of the International Society for Pharmacoeconomics and Outcomes Research (ISPOR) Task Force on Good RCT-CEA Research Practices and will use the sample size determined for primary outcome in Aim 1 for cost analyses [66, 67]. Unit of analysis will be the patient [68]. All costs will be discounted to the base year according to time elapsed since randomization [69]. Distribution of cost data will be examined, and the differences in costs between study groups will be tested accounting for the nesting of patients within team clusters. Generalized linear models for Poisson distribution, or zero-inflated Poisson or negative Binomial model for counts of different healthcare utilizations will be used to compare the groups. Each type of health service use (e.g., hospitalizations, emergency room visits) will be analyzed separately. Two-part models for expenditures will typically be adopted to account for zero-expenditure for some individuals [70].

##### Outcome assessment

From eCAP data, we construct an index of *medication adherence intensity* (MAI) at *t=* 6 and 12months from the amount of medication taken, relative to the total amount of medication prescribed. This is a fractional outcome with values in the range [0, 1], with value 1 for perfect adherence and value 0 for no adherence at all. In the baseline period, 3months prior, for which eCAP data will not be available, a proxy measure will be used. Construction of MAI: The period (0, *t*] is partitioned into intervals, *k*=1,…*K* where *t*_0_ = 0, *t*_*K*_ = *t*. In the interval (*t*_*k* − 1_, *t*_*k*_]we obtain the total prorated amount prescribed (*f*_*k*_) and the actual amount taken (*m*_*k*_). The adherence for this interval and for the period (0, *t*] is a time-weighted proportion [28].

##### Patient Activation Measure (PAM)

PAM is a continuous score on a 0–100 scale. Higher scores are associated with better activation [71, 72]. For MAI as continuous response, the beta regression model [73] with logit link posits the conditional mean and variance where *f*<1 is the under-dispersion. For PAM as continuous response, we will use a linear mixed model with mean and variance. Methods for the UKPDS risk score and patient engagement are entirely analogous. The structural forms of and are elicited from preliminary analyses of residuals [64]. Estimation of model parameters is via full maximum likelihood (ML), quasi-ML or restricted ML [64].

##### Moderators and mediators

By including terms for potential confounders (e.g., gender, education, race/ethnicity) in the covariate mix, we will assess whether their inclusion substantively changes the magnitude of the treatment-outcome effect by examining the change in the regression coefficients for study arms. We will characterize the study results for males and females and determine the impact of sex on the intervention uptake and outcomes. A simple mediation analysis posits that some 6-month outcomes (e.g., patient activation, provider-prescribing for secondary prevention of CVD) might influence the 12-month outcomes. We will initially adopt the systems-equation approach of Baron-Kenny [74] and MacKinnon et al. [75, 76] before examining more complex models for multiple mediating and confounding effects informed by directed acyclic graphs [77].

##### Cost and healthcare utilization

The Micro-costing Questionnaire will be used to collect data from the expense report, including cost-generating events not directly linked to a specific patient, to calculate total labor expenses (e.g., training costs), and non-labor expenses (e.g., supplies, educational materials, and administrative overhead) utilized by each group in the study. Cost-generating events will include (1) training providers to promote behavioral change, (2) distributing DSTs and educational materials, (3) providing continued technical support to providers and patients in text messaging use, and (4) administrative overhead for FQHCs, if applicable. The Health Services Utilization Form was developed and tested in a prior trial and captures health services utilization and patient out-of-pocket expense [78]. It gathers self-reported number of times the patient visits a specialist (e.g., dietitian, cardiologist), emergency room/urgent care center/hospital for DM or heart problems, and co-payment, deductibles, and other out-of-pocket expenses for DM or heart problems during the study period. Occurrences of healthcare utilization will be valued using the average cost in the Medical Expenditure Panel Survey for similar patients [79].

### Interim analyses {21b}

There are no interim analyses planned.

### Methods for additional analyses (e.g., subgroup analyses) {20b}

#### Subgroup analyses

We explore which subgroups of patients have benefited from the intervention through a cautious search [80, 81] of covariate patterns that contribute to “large” individual treatment effects. Statistical inference is not proposed. Using interaction terms between patient characteristics and intervention indicators, we will assess their impact on outcomes PAM and MAI at 12 months. A formal test of the interaction is not expected to be significant, but the size of the interaction might inform heterogeneity of the intervention effect.

### Methods in analysis to handle protocol non-adherence and any statistical methods to handle missing data {20c}

#### Addressing missingness [82]

In linear and nonlinear mixed models, likelihood-based inference using only the non-missing responses is valid under missing at random (MAR). Missing completely at random is likely to be untenable in our context. Techniques such as inverse-probability weighting will be used to accommodate missing data patterns that are neither MCAR nor MAR [83–85], an approach that we used in analyzing longitudinal censored medical costs data [85, 86].

### Plans to give access to the full protocol, participant-level data, and statistical code {31c}

Our tools and protocols will be available to the entire FQHC network and other health centers and systems to facilitate adoption and implementation of the program. We plan to disseminate findings via conference presentations at the National Association of Community Health Centers, Society of General Internal Medicine, American Heart Association (AHA), American Diabetic Association (ADA) Scientific meetings, other academic and public audiences and contribute to the AHRQ and CMS Innovation Web portal. Through the rigor and innovativeness of our proposed mixed methods assessment grounded in an integrated REAIM (Reach, Effectiveness, Adoption, Implementation, Maintenance) and Consolidated Framework for Implementation Research (CFIR) framework, the proposed research will contribute both to translational and implementation science. Finally, we will disseminate results in high impact peer-reviewed journals and present study findings at national professional meetings. Our results will be made available in the public domain. Likewise, tools and approaches that we generate will be openly shared without cost.

## Oversight and monitoring

### Composition of the coordinating center and trial steering committee {5d}

This is a multi-center study designed, implemented, and coordinated across 3 regions in the Midwest, USA.

Principle Investigator: supervises and oversees all aspects of the project.

Project Manager: responsible for day-to-day management of study sites, research assistants, and annual reports.

Research assistants: will recruit participants, conduct group visits and follow-up visits, train, and provide technical assistance to participants with the mobile phone platform Way to Health (W2H).

Biomedical Research Informatics Core (BRIC): organizes data collection and safeguards quality and data.

Research team: meets weekly.

There is no trial steering committee or stakeholder and public involvement group.

### Composition of the data monitoring committee, its role, and reporting structure {21a}

Data and safety monitoring plan: In compliance with NIH requirements, we established both a data and safety monitoring plan (DSMP) and formed a Data and Safety Monitoring Board (DSMB). The purpose of the DSMP and DSMB are to ensure the safety of participants and the validity and integrity of the data. The Independent DSMB (independent of the study investigators and sponsor) will oversee the safety of participants in each phase of the research strategy as needed, review adverse events in a timely fashion, and ensure that appropriate management is initiated and completed. Members of DSMB will be available for consultation for issues of ethics, clinical care, and human subject protection. The DSMB will identify adverse events and protocol deviation (e.g., violation of human subjects’ rights, and/confidentiality, compromised data integrity) and will quickly ensure appropriate reporting to the IRB and NHLBI. The membership consists of 4 members with appropriate expertise in clinical trials, biostatistics, cardiovascular disease, behavior interventions, and disparities research. This committee will meet twice a year via conference call or Zoom meeting and will report to the funding institute on scientific and administrative issues.

### Adverse event reporting and harms {22}

Although adverse events are not anticipated from the nature of this study, it is MSU IRB policy to provide all necessary care to study participants who experience adverse events during a clinical research study. The PI will submit yearly progress reports to the IRB and NHLBI with documentation of any significant issues. The following information will be included in the report: date of event, attribution to intervention, and outcome of adverse events. Death will be reported within 24 h. Unanticipated adverse events will be reported within 7 days. Reports will be submitted electronically to the MSU IRB and NIH. A written follow-up will be submitted within 30 days. All adverse events (serious or not serious, related, or unrelated, anticipated or unanticipated) will be reported in the annual report to MSU IRB and NIH.

### Frequency and plans for auditing trial conduct {23}

The Independent DSMB (independent of the study investigators and sponsor) will oversee the trial conduct and safety of participants in each phase of the research strategy. This committee will meet twice a year via conference call or Zoom meeting and will report to the funding institute on scientific and administrative issues.

### Plans for communicating important protocol amendments to relevant parties (e.g., trial participants, ethical committees) {25}

Important protocol modifications will be communicated to Institutional IRB and will be communicated to the sponsor, NHLBI/NIH as required by these relevant parties.

## Dissemination plans {31a}

### Dissemination plan

The study is registered at the ClinicalTrials.gov. as outlined in the policy and according to the specific timelines stated in the policy.

Our informed consent documents for the clinical trial will include a specific statement informing participants that we will be posting clinical trial information as required per NIH policy at ClinicalTrials.gov.

This study will comply with the Michigan State University Human Research Protection Program policy for ensuring clinical trial registration and results reporting.

### Dissemination plan in Michigan and other states

We will report our findings to the Michigan Primary Care Association (MPCA). MPCA is the voice of 45 community health centers that provide primary and preventive health care to more than 700,000 patients in rural and urban communities across Michigan. MPCA Health Centers are part of a nationwide network of 1200 Health Centers spread across 50 states and all US territories that provide care for over 29 million people in 9000 communities.

The results of this study will provide evidence for improved practices that can be directly incorporated into the community health centers. Additionally, FQHCs are all governed by a community board composed of 51 percent or more of Health Center patients who represent the population served; our work will be known by communities of patients who can raise awareness of successful approaches in their communities.

The Michigan Public Health Institute (MPHI) and the Michigan Department of Health and Human Services (MDHHS) provide additional avenues for dissemination to FQHCs in Michigan.

### Dissemination to the research community

Our tools and protocols will be available to the entire FQHC network and other health centers and systems to facilitate adoption and implementation of the program. We plan to disseminate findings via conference presentations at the National Association of Community Health Centers, Society of General Internal Medicine, American Heart Association (AHA), American Diabetic Association (ADA) Scientific meetings, and other academic and public audiences and contribute to the AHRQ and CMS Innovation Web portal. Through the rigor and innovativeness of our proposed mixed methods assessment grounded in an integrated REAIM (Reach, Effectiveness, Adoption, Implementation, Maintenance) and Consolidated Framework for Implementation Research (CFIR) framework, the proposed research will contribute both to translational and implementation science.

## Discussion

This randomized community-engaged pragmatic trial is designed to evaluate the efficacy and cost-effectiveness of the Office-GAP intervention with and without a mobile health reinforcement component. A key element of this pragmatic trial is to document challenges to implementation in our clinical sites, all Federally Qualified Health Centers in Michigan.

## Limitations

This trial includes seventeen care teams and takes place in multiple clinics across the state. Consistent and coordinated recruitment and enrollment of patients in the study is therefore a critical element of the trial. Research assistants on our team work closely with clinical staff to identify eligible patients and invite their participation into one of the two arms of the trial. In the rural part of the state, the clinics are spread quite far apart, requiring the RA to make visits to five different facilities across the county. In our urban centers in Lansing, high staff turnover has presented challenges for keeping staff and providers informed about ongoing recruitment and trained to participate in the protocol. While our design anticipated these complications, we did not foresee the additional challenges brought about by the COVID-19 pandemic.

Our planned start date of the trial was delayed in 2020 because our university IRB paused all research with human participants in March due to the COVID-19 pandemic. We were able to be back in the clinics and to start enrolling patients starting in the Fall of 2020. Remote work and travel restrictions caused us to shift our team meetings and provider training to online formats, which added flexibility but also took some additional preparation time. We have also had to limit the size of our patient activation group visits and schedule more of these to be sure that these can be done safely. Both modifications continue still today. Finally, the rate of primary care staff turnover has increased, so we have had to train new providers as new personnel are hired in the participating clinics.

## Strengths

This trial evaluates the use of a shared decision-making intervention, with and without mobile health reinforcement, implemented in Federally Qualified Health Centers to improve cardiovascular risk in DM patients with uncontrolled blood glucose. Our study aims to address the most severe risks of mortality for this patient population and to evaluate the efficacy of the Office-GAP program to address significant health disparities in cardiovascular outcomes among this patient population.

Office-GAP tools such as a checklist along with provider training and patient activation meetings, and mobile health text message reinforcement, all designed to be inexpensive and efficient to implement. We will evaluate and document any barriers implementation in the trial using the RE-AIM and CFIR frameworks. Cost-effectiveness will also be evaluated with an assessment of healthcare utilization.

## Trial status

Protocol Version 2 as of 11 5 2019

Recruitment started on May 26, 2021. The current protocol is version 2 as of 11 5 19. Patient recruitment is estimated to be completed around April 2024.

## Supplementary Information

**Additional file 1.**

## Data Availability

Data sharing: The datasets used and analyzed from this Office-GAP IMPACT study will be made available from the corresponding author upon reasonable request.

## Abbreviations

Office-GAP: Guidelines Applied to Practice
W2H: Way to Health
CVD: Cardiovascular disease
T2DM: Diabetes mellitus type 2
ACP: American College of Physician
ADA: American Diabetic Association
FQHCs: Federally Qualified Health Care Centers
RE-AIM: Reach, Effectiveness, Adoption, Implementation, Maintenance
CFIR: Consolidated Framework for Implementation Research
SDM: Shared Decision Making
DST: Decision Support Tool
PAI: Patient activation intervention
