# Supplementary material for: Improving diabetic patients’ adherence to treatment and prevention of cardiovascular disease (Office Guidelines Applied to Practice—IMPACT Study)—a cluster randomized controlled effectiveness trial
Source: Trials. 2022 Aug 15;23:659. doi: 10.1186/s13063-022-06581-6 (PMC9376908; doi:10.1186/s13063-022-06581-6)
Supplement: Supplementary file 1 — Additional file 1. [file 13063_2022_6581_MOESM1_ESM.pdf]

# Research Participant Information and Consent Form

You are being asked to participate in a research study. Researchers are required to provide a consent form to inform you about the research study, to convey that participation is voluntary, to explain risks and benefits of participation, and to empower you to make an informed decision. You should feel free to ask the researchers any questions you may have.

**Study Title: Improving Diabetic Patients' Adherence to Treatment and Prevention of Cardiovascular Disease**


**Researcher and Title**: Ade Olomu MD, MS, FACP: Swartz Endowed Professor of Medicine
**Department and Institution**: Department of Medicine, Michigan State University
**Address and Contact Information**: Department of Medicine, Michigan State University,
788 Service Road, B319 Clinical Center
Telephone: 517-884-7891
Fax: 517-432-1326


**Sponsor:** National, Heart, Lung and Blood Institute, National Institutes of Health


## 1. PURPOSE OF RESEARCH

- You have been selected as a possible participant in this study because you are receiving treatment for diabetes.
- We hope to learn whether patient participation in shared decision-making with their provider, and the use of text messages to follow up patients' care can lead to better medication use and better control of patients' diabetes and ultimately help to decrease the burden of disease and chronic illness.
- We hope to also learn if reminders to providers can help them consistently recommend medicines, diet, exercise and smoking cessation to patients.
- Your participation in this study will take about 12 months.

## 2. ALTERNATIVE OPTIONS

If you decide not to take part in this research study, you should know that it will not make any difference in the quality of any medical care or treatment you may receive from your provider.

## 3. WHAT YOU WILL DO

If you agree to participate in the study, Dr. Ade Olomu and her associates may give you:

a) 1-page *Office-GAP (Guidelines Applied to Practice) Checklist:* It will list all the medications that patients with diabetes should be taking and the lifestyle changes that they should be practicing to prevent heart disease. You will be asked to check whether you are taking each medication currently. If not, you will be asked to write down why you are not taking the medication. The same will be done with lifestyle.

You and your provider will sign the Office-GAP Checklist. It will help you and your provider to be clear about what you should do. You will be given a copy of the Checklist to take home.

b) List of programs in your community that can assist you in making necessary lifestyle changes. For example, where you can get dietary advice and help with your weight management or help to quit smoking, or how to obtain your medications.

c) Other types of educational materials.

d) You will receive text messages about your medications, or taking care of your diabetes (e.g. Time to check your blood sugar /blood pressure) and some questions (such as, “do you need any refills of any of your medications?”) to which you may respond by texting.

e) This is an educational intervention. You may receive educational information that will focus on diabetic self-care/ taking your medications (such as taking your medications regularly as prescribed, information on nutrition, glucose monitoring, foot care, and exercise) and one topic relating to living with chronic illness (such as navigating the healthcare system and managing stress).

f) Brief surveys before and after the study about:
   - How good the GAP programs are (if you were given the GAP Checklist).
   - Your confidence about doing what you decided.
   - Your active participation in your care.
   - How satisfied you are with how the providers explained things to you.

g) You may be contacted to participate in an exit interview about your experience with the study.

h) For this project you will attend a one-time group visit that will take 90-120 minutes. After that you will attend follow up visits with your provider at 0-1, 3, 6, 9 and 12 months that will be dedicated to prevention of heart disease.

This project **<u>does not</u>** involve any experimental drugs or invasive medical procedures.

- The patients in the study will be assigned by random, that is, by a method of chance, to one of two groups:
  Group 1: Will receive text messages as described in items d and e above, plus receive the Office GAP Checklist as described in item a above.
  Group 2: Will receive only text messages as described in items d and e above.

- You will have an equal chance of being in either group of the study.

## 4. POTENTIAL BENEFITS

The potential benefit to you for taking part in this study is that you may 1) learn better ways

to help you improve the use of your medications and lifestyle changes that are known to help prevent heart attacks, 2) experience better control of your diabetes, blood pressure and cholesterol during the study, 3) learn how to manage your chronic conditions and engage in shared decision making with your provider, and 4) Your participation in the study may help us to learn whether the use of text-messages or patient engagement can help to improve the care we provide to all patients in general and lead to a better outcome for them.

## 5. POTENTIAL RISKS

This study poses no known additional risk to patients or providers. The goal is to improve use of prescribed medications you are already taking for better control of your diabetes. We do not anticipate that the research activities will be associated with higher rates of low blood pressure/high blood pressure, low HbA1c/high HbA1c compared to the usual care. However, it is possible that as a result of increased monitoring from the program and text messages, we will detect more episodes of elevated blood pressure, cholesterol or HbA1c. Patients will be told by Research Assistants (RA) under supervision of Dr. Ade Olomu to contact their provider for immediate intervention and management of any abnormal blood pressure, cholesterol or HbA1c. You can withdraw from the study at any time and it will not make any difference in the usual care you receive from your provider.

## 6. PRIVACY AND CONFIDENTIALITY

The information collected about you during the study will be kept confidential to the maximum extent allowable by law. Your privacy will be protected at all times. All identifiable study data will be stored in a locked cabinet by the Research Assistant in Alpena, Lansing or Saginaw. There are 3 distinct ways in which study participants' informed consent, HIPAA and surveys from the 12 clinical sites are brought in and filed in the Research Office at Clinical Center, Room B319.

I: Research assistants will mail informed consent, HIPAA and survey documents via U.S. mail to Clinical Center, Room B319. The documents will be stored in a locked cabinet in Room B319 at the Clinical Center.

II: Informed consent, HIPAA, and survey documents will be transported by vehicle to Clinical Center, Room B319 in a secure case which is not readily visible. The documents and surveys will be stored in a locked cabinet in Room B319 at the Clinical Center.

III: Study surveys will be transported by vehicle, in a secure case which is not readily visible, to other Study home office (Alpena, Lansing or Saginaw) for the purpose of verifying survey data entry. Once verified, surveys will be transported by vehicle in a secure case to Clinical Center, Room B319.

All study information will be kept on an MSU Health Information Technology password protected server or Cloud technology. The study information and consent forms will be secured until all study reports and manuscripts have been completed. The study information/data and all forms and surveys for the study will be kept for 3 years following closure of the study. Then they will be destroyed. Only designated study team members, MSU Human Research Protection Program (HRPP) and the Sponsor (National Institute of Health) will ever be provided access to any study research data by the principal investigator, Dr.

Olomu.

This research is covered by a Certificate of Confidentiality from the National Institutes of Health. This means that the researchers cannot release or use information, documents, or samples that may identify you in any action or suit unless you say it is okay. They also cannot provide them as evidence unless you have agreed. This protection includes federal, state, or local civil, criminal, administrative, legislative, or other proceedings. There are some important things that you need to know. The Certificate does not stop reporting that federal, state or local laws requires. Some examples are laws that require reporting some communicable diseases and threats to harm yourself or others. Researchers may release information about you when you say okay. For example, you may give them permission to release information to insurers, medical providers or any other persons not connected with the research. The Certificate of Confidentiality does not stop you from willingly releasing information about your involvement in this research. It does not prevent you from having access to your own information.

There may be results of the research that may be relevant to your clinical care. You will be told if your Blood Pressure, HbA1c, or cholesterol values are too high or too low and your provider will be informed as well. The results of this study may be published or presented at professional meetings, but no research participants will not be identified in any way as a result of this research. Your confidentiality will be protected to the maximum extent allowable by law.

## 7. YOUR RIGHTS TO PARTICIPATE, SAY NO, OR WITHDRAW

Participation in this research project is completely voluntary. You have the right to say no. You may change your mind at any time and withdraw. You may choose not to answer specific questions or to stop participating at any time. Choosing not to participate or withdrawing from the study will not make any difference in the quality of any medical care or treatment you may receive from your provider.

## 8. COSTS AND COMPENSATION FOR BEING IN THE STUDY

For your participation in this study, there will be no cost for you or your insurance provider for the group visit. You will receive $20 for attending the group visit. The 5 follow up visits will be regular office visits with your provider, but the focus will be on the prevention of heart disease. You will be compensated $20, $20, $20, $20, $50, (total of $150) for attending 0-1, 3, 6, 9, 12 month follow up visits. Patients receiving Office-GAP + Texting will receive $50 more for additional time spent (total of $200). Patients that participate in an exit interview will receive an additional $40. You will be responsible for any insurance co-pays during the follow up appointments with your provider.

## 9. FUTURE RESEARCH

Your medical records information that includes your medical history, physical examination findings, laboratory results, radiology or echocardiogram or any information obtained through the surveys as part of the research, will only be stored or shared with NIH, in accordance with

NIH Policies for future research studies. MSU HRPP may have access to these records. However, no research participants will be identified in any way as a result of this research.

**10. CONTACT INFORMATION**

If you have concerns or questions about this study, such as scientific issues, how to do any part of it, or to report an injury, please contact the researcher Dr. Ade Olomu, MD, MS, FACP at B323 Clinical Center, Department of Medicine Michigan State University, East Lansing, MI, 48824, 517- 432-0897.

If you have questions or concerns about your role and rights as a research participant, would like to obtain information or offer input, or would like to register a complaint about this study, you may contact, anonymously if you wish, the Michigan State University's Human Research Protection Program at 517-355-2180, Fax 517-432-4503, or email irb@msu.edu or regular mail at 4000 Collins Rd. Lansing, MI 48910.

**11. DOCUMENTATION OF INFORMED CONSENT**

Your signature below means that you voluntarily agree to participate in this research study.

Participant's Name:____________________________________________

Date: ______________________________

______________________________
Signature

Person Obtaining Consent: Name______________________________________

Date: ______________________________

______________________________
Signature

You will be given a copy of this form to keep.
